# Obstacles Deterring Medical Students From Pursuing a Career in the Field of Surgery in Jazan University, Saudi Arabia

**DOI:** 10.7759/cureus.43233

**Published:** 2023-08-09

**Authors:** Nasser Hakami, Abdullah Madkhali, Fatimah Hakami, Maryam ALshekh, Enas Masmali, Dalal Hamithi, Basem Zogel

**Affiliations:** 1 Faculty of Medicine, Jazan University, Jazan, SAU

**Keywords:** jazan, surgical, career, obstacles, determine

## Abstract

Background: Particularly in Saudi Arabia, there is a dearth of trained specialists in the field of surgery. Understanding the obstacles that discourage medical students and residents from pursuing a surgical career is essential for resolving this shortage. This study intended to investigate the characteristics that influence medical students and trainees at Jazan University, Saudi Arabia, to pursue a career in surgery.

Methodology: This observational study employed a descriptive, cross-sectional approach. The intended audience consisted of fourth- to sixth-year medical students and medical residents. The questionnaire gathered information on demographics, academic year, previous surgical experience, perceptions of surgery as a specialty, and variables influencing career selections.

Results: Out of 413 participants, 74.3% were considering a surgical career, with 24.4% interested in general surgery, followed by cardiac surgery (14.3%) and pediatric surgery (12.4%). Factors influencing career decisions included potential income (82%), the possibility of part-time work (82%), and partial leave (74%). A significant proportion of participants agreed or strongly agreed that the incidence of suicide (62%) and the risk of depression (72%) are higher in surgical specialties. Female participants were more likely to agree that their chance of becoming a spouse could be affected by a surgical career (p=0.002) and that meeting role models could influence their choice of surgical specialty (p=0.015).

Conclusion: Work-life balance, long working hours, and mental health concerns are identified as variables that discourage medical students and residents from pursuing a surgical career in the study. Efforts to encourage work-life balance, minimize workload, and provide support and resources for mental health issues should help surgeons suffer less stress and burnout. Moreover, encouraging an open-minded attitude and de-stigmatizing mental health concerns in the medical field should encourage individuals to seek assistance when necessary and lower the incidence of suicide and depression. Finally, tackling gender discrimination and encouraging diversity and inclusion in surgical specialties could attract more skilled surgeons.

## Introduction

The choice of postgraduate specialties has changed over time, and medical students begin their studies with a career plan [1-3]. The decision to specialize is the result of a complicated interplay between a number of variables, including student interests and expectations, future use of the specialty, the number of openings in the job market at the time, advice from professionals, suggestions from family, and societal influences. There has been growing worry over the past ten years about a drop in the number of medical students who want to work in the field of surgery [4]. This is supported by a number of international studies, particularly those from South Africa and the United States [5-7]. It is important to understand the barriers that prevent both men and women in the medical profession from choosing surgery as their specialty and the research opportunities provided to them by their mentors and their decision to pursue surgery as a career in college [8-9]. At the same time, the demanding and long hours of work diminish the attractiveness of the specialty to students. These difficulties exacerbate emotional and physical exhaustion and frequently discourage junior doctors and medical students from choosing a career in surgery. Given the accelerated population growth and the concomitant decline in the number of surgeons, it was imperative to address this problem. A large proportion of studies addressing the reasons for choosing a surgical profession have been limited to high-income countries [10]. The literature has called for a renewed awareness of surgery among medical students, as the proportion of women and graduate students entering medical school is increasing while the likelihood of them choosing a surgical career is decreasing [11-12]. In addition, previous studies have shown that students' choices were influenced by their gender status and the unsavory lifestyle of the surgeon. According to a study from Germany, only 5.6% of female medical students choose surgery, compared with 15.2% of their male peers. Since 70% of medical students are female, this may discourage them from pursuing a surgical career [13-14]. The surgical workforce will be in short supply in the future. It is important to discover the elements that influence the students' choices and to use the data to provide adjustments to the system when appropriate, given that the opinions and experiences shared throughout undergraduate training influence the students' future career decisions [15-16]. There are few studies on the challenges faced by medical students who choose to pursue a surgical career in Saudi Arabia. Accordingly, this study aims to evaluate the different barriers and obstacles facing Saudi medical students when choosing a surgical specialty [17].

There is a paucity of research exploring factors influencing the decision to pursue a surgical career, and a large portion of the literature examining reasons why people choose to become surgeons only considers situations in high-income countries. To better understand the challenges and obstacles in choosing a surgical profession, which might enhance and boost student recruitment into academic surgical careers, we created a cross-sectional survey aiming to identify barriers to pursuing a surgical career at Jazan University, Saudi Arabia. Therefore, the current study aimed to identify obstacles deterring males and females from pursuing a career in the surgical field at Jazan University, Saudi Arabia.

## Materials and methods

The study was a descriptive, cross-sectional observational design that aimed to explore the barriers that deter male and female medical students from pursuing a career in the surgical field at Jazan University, Saudi Arabia. The target population was medical students from the fourth to sixth year of study and medical trainees, including house officers and residents aged between 18 and 40 years. The study excluded surgical trainees or residents who had already selected surgery as a career.

Data were collected from March to May 2023 by sending an electronic questionnaire to Jazan University medical students from the second year and above, as well as medical trainees. The questionnaire was conveniently distributed from the alumni database of Jazan University around Saudi Arabia. The sample size was calculated using the formula n=z2(1-)P(P-1)/d2, where n is the sample size, z is the confidence level, P is the estimated proportion, and d is the desired precision. Assuming a 95% confidence level, 50% estimated proportion, and 5% desired precision, the sample size was 364 participants, with a 10% refusal rate.

The questionnaire included questions related to demographic information, academic year, past surgical experience, perceptions of surgery as a specialty, and factors influencing career decisions. The questionnaire was collected anonymously, and the data were analyzed using SPSS software version 26 (IBM Corp., Armonk, NY). Descriptive statistics were used to summarize the data, including means, standard deviations, frequencies, and percentages. The chi-square test was used to explore the statistical significance of differences between groups, and the t-test was used to compare the main outcome variables and age.

Ethical approval was obtained from Jazan University and the Jazan Committee, and informed consent was obtained from all participants before filling out the questionnaire. The data were stored under strict confidentiality levels to protect participants' privacy. The study aimed to provide evidence for decision-makers to implement interventions such as screening and educational programs to address the barriers deterring medical students from pursuing a career in surgery.

## Results

The demographic factors of the participants are presented in Table 1. A total of 413 participants took part in the study, with 58.4% being female and 41.6% being male. The majority of participants (87.9%) were aged between 18 and 25 years, with 11.6% aged between 26 and 30 years and only 0.5% aged between 31 and 35 years. Regarding academic year or work, the majority of participants were in their fourth year of study (53.8%), followed by the fifth year (19.9%), and sixth year (16.0%). A small percentage of participants were interns (3.9%), while residents in various stages of their training accounted for 6.1% of the sample, with the majority being R1 (4.4%). Only 1.0% of participants were specialists.

**Table 1:** Demographic factors of the participants

		Frequency	Percentage (%)
Gender	Male	172	41.6
	Female	241	58.4
Age	18-25	363	87.9
	26-30	48	11.6
	31-35	2	0.5
Academic year or work	fourth year	222	53.8
	fifth year	82	19.9
	sixth year	66	16.0
	Intern	16	3.9
	Resident (R1)	18	4.4
	Resident (R2)	2	0.5
	Resident (R4)	1	0.2
	Resident (R6)	1	0.2
	Resident (R7)	1	0.2
	Specialist	4	1.0

Table 2 presents the participants' attitudes toward a surgical career. Out of the 413 participants, 307 (74.3%) were considering a surgical career, while 106 (25.7%) were not considering it. It was found that a significantly higher percentage of males would choose a surgical career than females (82% vs. 68.9%, P=0.003). Among those who were considering a surgical career, 24.4% were interested in general surgery, followed by cardiac surgery (14.3%) and pediatric surgery (12.4%). Neurosurgery (11.4%), plastic surgery (10.1%), and orthopedic surgery (7.8%) were also popular choices. Only a small percentage of participants were interested in urology surgery (1.0%). In contrast, among the 106 participants who were not considering a surgical career, the most commonly considered specialties were family medicine (33.3%) and internal medicine (23.8%). Of the participants who were residents, 36.7% were currently working in the surgical field, followed by critical care (12.2%) and ENT (ear, nose, and throat) (11.1%). Pediatrics (8.9%), ophthalmology (6.7%), and dermatology (5.6%) were also represented, while a smaller percentage of residents worked in internal medicine (3.3%), anesthesia (3.3%), emergency medicine (4.4%), psychiatry (3.3%), and family medicine (4.4%).

**Table 2:** Attitude of the participants toward a surgical career ENT, ear, nose, and throat

		Frequency	Percentage (%)
Are you considering any surgical career?	No	106	25.7
	Yes	307	74.3
If you are considering surgery, about which surgical sub-specialty you are thinking?	General Surgery	75	24.4
	Neurosurgery	35	11.4
	Pediatric Surgery	38	12.4
	Obstetrics and Gynecology	18	5.9
	Plastic Surgery	31	10.1
	Cardiac Surgery	44	14.3
	Ophthalmic Surgery	21	6.8
	Otolaryngology (ENT)	18	5.9
	Orthopedic Surgery	24	7.8
	Urology Surgery	3	1.0
If your considered specialty is not here, please write here your considered specialty.	internal Medicine	5	23.8
	Family Medicine	7	33.3
	Psychiatry	2	9.5
	Pediatric	4	19.0
	Anesthesia	1	4.8
	Dermatology	2	9.5
If you are a resident, please select your current job specialty.	Pediatrics	8	8.9
	Surgery	33	36.7
	Ophthalmology	6	6.7
	Internal Medicine	3	3.3
	Dermatology	5	5.6
	Critical Care	11	12.2
	ENT	10	11.1
	Family Medicine	4	4.4
	Emergency Medicine	4	4.4
	Psychiatry	3	3.3
	Anesthesia	3	3.3

The majority of participants (87%) agreed or strongly agreed that life balance could be affected by working as a surgeon. Similarly, 85% of participants agreed or strongly agreed that long working hours could influence their career choice. Over 86% of participants agreed or strongly agreed that surgery could affect their lifestyle. A significant proportion of participants also agreed or strongly agreed that gender discrimination in surgical specialties is common (72%) and that surgical specialties require courage (85%). Participants were less likely to agree or strongly agree that their chance of becoming a spouse could be affected (72%) or that they would be embarrassed during a rectal examination (70%). The majority of participants (68%) also agreed or strongly agreed that meeting role models could influence their choice of surgical specialty. Regarding career-related factors, 82% of participants agreed or strongly agreed that potential income could influence their career choice. A similar percentage agreed or strongly agreed that the possibility of part-time work (82%) or partial leave (74%) could also influence their choice of specialty. Finally, regarding mental health concerns, a significant proportion of participants agreed or strongly agreed that the incidence of suicide (62%) and the risk of depression (72%) are higher in surgical specialties (Table 3).

**Table 3:** Obstacles to choosing a surgical career according to the participants

	Strongly disagree		Disagree		Agree		Strongly Agree	
	Count	Row N	Count	Row N	Count	Row N	Count	Row N
You consider surgery to be suitable for you as 1 or a 2?	17	4.1%	37	9.0%	175	42.4%	184	44.6%
Life balance could be affected by working as a surgeon?	6	1.5%	26	6.3%	210	50.8%	171	41.4%
Long working hours influence my career choice?	6	1.5%	52	12.6%	189	45.8%	166	40.2%
Surgery will affect your lifestyle?	9	2.2%	46	11.1%	172	41.6%	186	45.0%
Gender discrimination in surgical specialties is common?	12	2.9%	101	24.5%	165	40.0%	135	32.7%
Surgical specialty needs courage?	5	1.2%	58	14.0%	182	44.1%	168	40.7%
Your chance of becoming a spouse could be affected?	22	5.3%	94	22.8%	170	41.2%	127	30.8%
Surgery is a male-dominant specialty?	37	9.0%	111	26.9%	148	35.8%	117	28.3%
You will be embarrassed during the rectal examination?	40	9.7%	83	20.1%	169	40.9%	121	29.3%
Meeting a female role model will influence my choice of surgical specialty?	25	6.1%	108	26.2%	176	42.6%	104	25.2%
You have been encouraged to choose your specialty?	27	6.5%	81	19.6%	193	46.7%	112	27.1%
Length of surgical specialties residency is reasonable?	18	4.4%	61	14.8%	213	51.6%	121	29.3%
Potential income influences my career choice?	17	4.1%	59	14.3%	179	43.3%	158	38.3%
Possibility of parental leave influences my career choice?	16	3.9%	89	21.5%	193	46.7%	115	27.8%
Possibility of part-time work influences my career choice?	12	2.9%	62	15.0%	198	47.9%	141	34.1%
The incidence of suicide in surgical specialties is increasing?	28	6.8%	129	31.2%	168	40.7%	88	21.3%
Surgical specialties increase the risk of depression?	25	6.1%	90	21.8%	180	43.6%	118	28.6%

Table 4 presents the differences in the perceptions of male and female participants regarding the obstacles to choosing a surgical career. The table shows the mean and standard deviation for each obstacle, separately for male and female participants, as well as the p-value for the difference between the two groups. Overall, male and female participants had similar perceptions of most obstacles. There were no significant differences between the two groups in their perceptions of life balance, long working hours, lifestyle, courage, dominance of surgery, embarrassment during rectal examination, encouragement to choose a specialty, length of residency, potential income, partial leave, and risk of depression. However, there were significant differences in the perceptions of male and female participants regarding two obstacles. Female participants were more likely to agree that their chance of becoming a spouse could be affected by a surgical career (p=0.002) and that meeting role models could influence their choice of surgical specialty (p=0.015) compared to male participants.

**Table 4:** Difference between the two genders in their perception of the obstacles against choosing a surgical career SD, standard deviation

	Gender					Total	
	Male		Female				
	Mean	SD	Mean	SD	P-value	Mean	SD
Life balance could be affected by working as a surgeon?	3.67	1.10	3.78	1.12	0.34	3.74	1.11
Long working hours influences my career choice?	3.59	1.15	3.69	1.19	0.41	3.65	1.17
Surgery will affect your lifestyle?	3.78	1.17	3.72	1.23	0.58	3.75	1.20
Gender discrimination in surgical specialties is common?	3.24	1.31	3.43	1.19	0.12	3.35	1.24
Surgical specialty needs courage ?	3.63	1.19	3.66	1.18	0.76	3.65	1.18
Your chance of becoming a spouse could be affected?	3.05	1.30	3.44	1.21	0.002*	3.28	1.26
Surgery is a male-dominant specialty ?	3.10	1.33	3.13	1.32	0.86	3.12	1.32
You will be embarrassed during rectal examination?	3.08	1.28	3.27	1.33	0.15	3.19	1.31
Meeting female role model will influence my choice of surgical specialty?	2.95	1.19	3.24	1.24	0.015*	3.12	1.22
You have been encouraged to choose your specialty?	3.19	1.22	3.23	1.23	0.74	3.22	1.22
Length of surgical specialties residency is reasonable?	3.38	1.13	3.33	1.21	0.70	3.35	1.17
Potential income influences my career choice ?	3.65	1.24	3.46	1.25	0.15	3.54	1.24
Possibility of parent leave influences my career choice?	3.23	1.14	3.29	1.23	0.65	3.26	1.19
Possibility of part-time work influences my career choice?	3.52	1.20	3.44	1.18	0.48	3.47	1.19
The incidence of suicide in surgical specialties is increasing?	2.92	1.19	3.02	1.21	0.39	2.98	1.20
Surgical specialties increase the risk of depression?	3.23	1.27	3.23	1.23	1.00	3.23	1.25

## Discussion

Numerous studies have proposed logical causes for the gender gap in the surgical field. Both male and female medical school students prioritize occupations that provide for a healthy work-life balance [18-21]. The purpose of this study was to examine the attitudes and beliefs of medical students and residents regarding a surgical career, as well as the demographic factors that may influence their career choice. Most of the study participants were in their fourth year of study and were between the ages of 18 and 25. Additionally, 74.3% of participants were considering a career in surgery, with men more inclined than women to pursue this path. This is similar to the findings of a number of prior studies, which demonstrated that female medical students are significantly less likely to choose a surgical specialty [19,22]. The most often considered surgical specializations were general surgery, heart surgery, and pediatric surgery. Several studies have given plausible explanations for this gender discrepancy, such as the nature of the surgical practice, the presence of positive role models, and the perception of high job satisfaction [23-24].

An intriguing finding of the study was that the majority of participants agreed or strongly agreed that working as a surgeon could impair life balance, lengthy working hours, and lifestyle. This emphasizes the significance of work-life balance in the medical field, especially in high-stress professions such as surgery [25]. Medical schools and residency programs must address this issue and assist their students and residents in achieving a healthy work-life balance.

The impression of gender discrimination in surgical specialties was another noteworthy conclusion of the study. The majority of respondents agreed or strongly agreed that gender bias is prevalent in surgical specialties. Multiple studies have uncovered indications of gender discrimination and bias in surgical specialties. A 2020 study, for instance, indicated that female surgeons experienced prejudice in the form of being treated differently and hearing comments about their sex [26]. In addition, research has revealed that female surgeons encounter obstacles such as a lack of mentorship, income discrepancies, and work-life balance concerns [27]. Sexual harassment and discrimination have been highlighted as significant impediments to surgical residency and fellowship training [28]. In addition, a survey done in 2020 revealed that female medical students are significantly less likely to choose a surgical specialty, possibly due to perceived constraints such as long workdays, limited leisure time, and societal or cultural obstacles [24]. While progress has been made in the fight against gender disparity in surgery, more must be done to guarantee that women have equal opportunities to seek and succeed in surgical careers [29]. This emphasizes the need for measures to overcome gender disparity in the medical field, particularly in surgical specialties where the gender gap is still pronounced. Medical schools and residency programs should seek to provide all students and residents, regardless of gender, with equitable opportunities.

In addition, the study indicated that prospective pay, the option of a part-time job, and partial leave could impact participants' choice of specialty. For instance, medical students and residents who value work-life balance may be more inclined to pick specialties that allow more flexible work arrangements, such as part-time or reduced hours [30]. Similarly, if a specific surgical subspecialty offers a greater income or better benefits, this may also attract medical students and residents [31]. It is essential to highlight, however, that other considerations, such as intellectual challenge, mentorship, and exposure to a variety of specialties, may also play a substantial role in the decision-making process [32]. This emphasizes the significance of financial factors in employment choices. To assist students and residents in making educated career selections, medical schools and residency programs must disclose information regarding potential income and work arrangements in various specialties.

Concerning mental health issues, a sizeable majority of participants agreed or strongly agreed that the frequency of suicide and the risk of depression are greater in surgical specialties. Compared to the general population and other medical specializations, surgeons have a higher risk of depression, burnout, and suicide, according to multiple studies [33-35]. This may be partially attributable to the rigorous and high-pressure nature of the surgical profession, which can result in extended work hours, high levels of stress, and an imbalance between work and personal life [36]. In addition, surgical residents and fellows may encounter sleep deprivation, financial problems, and the pressure to perform well on tests and procedures with high stakes. A systematic scoping review conducted in 2022 demonstrated how various barriers hinder career progression in surgery for women, with clear implications for burnout and attrition. The focus of 22 (18%) of the articles included was on issues affecting the choice of a surgical career, such as long surgical workdays and little free time for outside interests. Fifty-five (46%) analyzed the major obstacles to surgical residency and fellowship training, such as sexual harassment and discrimination. Twenty-seven (23%) of the articles discussed obstacles to professional progression, such as overwhelming workloads, insufficient mentoring, ambiguous expectations for advancement, salary disparities, or issues with work-life balance [28]. This underlines the importance of addressing mental health issues in the medical field, particularly in high-stress professions such as surgery. Medical schools and residency programs should give students and residents access to mental health services and resources to assist them in maintaining excellent mental health.

In addition, the study revealed substantial disparities in the beliefs of male and female participants regarding two obstacles: the impact on their likelihood of becoming spouses and the impact of meeting role models on their choice of surgical specialty. Female participants were more likely to agree that a surgical career could affect their likelihood of becoming a spouse, which may reflect the gendered expectations and societal norms that persist in some societies. In addition, female participants were more likely to think that meeting role models could impact their choice of surgical specialty, which may reflect the significance of representation and mentoring for women entering male-dominated industries like surgery.

## Conclusions

The present study offers significant insights into the attitudes and beliefs of medical students and residents regarding a surgical career. The research emphasizes the significance of work-life balance, gender equality, financial considerations, and mental health assistance in career choice. The findings also show the need to address gendered expectations and equip women entering surgical specialties with representation and mentorship. These findings can help in the efforts of medical schools and residency programs to assist students and residents in making informed career decisions and to promote a diverse and inclusive medical profession.
